# Characterization of CurcuEmulsomes: nanoformulation for enhanced solubility and delivery of curcumin

**DOI:** 10.1186/1477-3155-11-37

**Published:** 2013-12-06

**Authors:** Mehmet H Ucisik, Seta Küpcü, Bernhard Schuster, Uwe B Sleytr

**Affiliations:** 1 Department of Nanobiotechnology, Institute for Synthetic Bioarchitectures, University of Natural Resources and Life Sciences (BOKU) Vienna, Muthgasse 11, Vienna 1190, Austria; 2 Department of Nanobiotechnology, Institute for Biophysics, University of Natural Resources and Life Sciences (BOKU) Vienna, Muthgasse 11, Vienna 1190, Austria

**Keywords:** Curcumin, Emulsome, Nanocarrier, Lipophilic drug, Cell cycle arrest, Drug delivery, Enhanced solubility, Prolonged release, Nanoformulation

## Abstract

**Background:**

Curcumin is a polyphenolic compound isolated from the rhizomes of the plant Curcuma longa and shows intrinsic anti-cancer properties. Its medical use remains limited due to its extremely low water solubility and bioavailability. Addressing this problem, drug delivery systems accompanied by nanoparticle technology have emerged. The present study introduces a novel nanocarrier system, so-called CurcuEmulsomes, where curcumin is encapsulated inside the solid core of emulsomes.

**Results:**

CurcuEmulsomes are spherical solid nanoparticles with an average size of 286 nm and a zeta potential of 37 mV. Encapsulation increases the bioavailability of curcumin by up to 10,000 fold corresponding to a concentration of 0.11 mg/mL. Uptaken by HepG2 human liver carcinoma cell line, CurcuEmulsomes show a significantly prolonged biological activity and demonstrated therapeutic efficacy comparable to free curcumin against HepG2 *in vitro* - with a delay in response, as assessed by cell viability, apoptosis and cell cycle studies. The delay is attributed to the solid character of the nanocarrier prolonging the release of curcumin inside the HepG2 cells.

**Conclusions:**

Incorporation of curcumin into emulsomes results in water-soluble and stable CurcuEmulsome nanoformulations. CurcuEmulsomes do not only successfully facilitate the delivery of curcumin into the cell *in vitro*, but also enable curcumin to reach its effective concentrations inside the cell. The enhanced solubility of curcumin and the promising *in vitro* efficacy of CurcuEmulsomes highlight the potential of the system for the delivery of lipophilic drugs. Moreover, high degree of compatibility, prolonged release profile and tailoring properties feature CurcuEmulsomes for further therapeutic applications *in vivo*.

## Background

Curcumin, chemically known as diferuloyl methane, is a hydrophobic polyphenol derived from the rhizome of the plant *Curcuma longa* (turmeric) of the Zingiberaceae family. Curcumin is known to suppress multiple signaling pathways and inhibit cell proliferation, invasion, metastasis and angiogenesis [1]. Its wide medical use includes anti-septic, analgesic, anti-inflammatory, anti-oxidant, anti-malarial and wound-healing [2]. In recent years, a particular interest was shown on the anti-oxidative and anti-inflammatory properties of curcumin which might provide a therapeutic window for cancer treatment [3].

Curcumin is a yellow-colored tautomeric compound that is quite soluble in organic solvents such as dimethoxy sulfoxide (DMSO), ethanol, methanol, chloroform or acetone. Upon dissolution in an organic solvent, curcumin absorbs light in the visible wavelength range [4]. Turmeric contains three major analogues: curcumin, demethoxycurcumin (DMC), and bisdemethoxycurcumin (BDMC) and recently identified cyclocurcumin in less significant amounts [5]. Commercially available curcumin mixture contains approximately 77% curcumin, 17% DMC and 3% BDMC as major components [6]. Although all three are highly active, curcumin is more efficient than DMC and BDMC on various cell models [6,7]. Contrary to these findings, studies on preclinical models of carcinogenesis have demonstrated that commercial grade curcumin – turmeric as a mixture - has the same inhibitory effect as pure curcumin [8,9].

Pharmacologically regarded as safe, curcumin is nontoxic, even at relatively high doses [10] such as 8 g per day [11]. As demonstrated recently, tumor cells are more sensitive to the cytotoxic activity of curcumin than normal cells [12]. In line with another study, the cellular uptake of curcumin was found to be significantly higher in tumor cells compared to normal cells, which was attributed to the differentiated membrane structure, protein composition and bigger size [13]. The lower uptake rate may explain the low toxicity of curcumin for healthy cells.

The wide spectrum of pharmacological properties of curcumin is attributed to its numerous effects on several targets including transcription factors, growth regulators, adhesion molecules, apoptotic genes, angiogenesis regulators, and cellular signaling molecules [14]. Curcumin exerts anti-cancer activity mainly through blocking cell cycle progression and triggering tumor cell apoptosis [15]. All three stages of carcinogenesis including initiation, promotion and progression are suppressed by curcumin [16]. This is probably due to inhibition of the nuclear factor κB, which plays a central role in regulating the expression of various genes involved in cell survival, apoptosis, carcinogenesis and inflammation. This efficacy makes curcumin to a potential therapeutic target [17]. Furthermore, curcumin affects various cell cycle proteins and checkpoints involving downregulation of some of the cyclins and cyclin-dependent kinases (cdk), upregulation of cdk inhibitors, and inhibition of DNA synthesis [18]. However, the physiological response triggered by curcumin depends on the cell type, the concentration of curcumin (IC_50_: 2-40 μg/ml) and the time of treatment [19]. For instance, curcumin treatment was reported to arrest cell growth at G2/M phase and induce apoptosis in human hepatoma cell line HepG2 [20,21], whereas G0/G1 as well as G1/S phase arrests were reported for various other cell lines [18].

Clinical use of curcumin remains very limited due to its extremely poor water solubility (≈11 ng/ml) [22], and low bioavailability following oral administration [23]. Even when 10-12 g/ml of curcumin was administered orally in humans, curcumin levels in serum remained approximately at 50 ng/ml [24]. Several studies demonstrated that 10-50 μM (3.7-18.4 μg/ml) curcumin induces cell death primarily through apoptosis [25,26]. However, the important question to be addressed is how to bring curcumin at these micromolar concentrations to the site of tumors while curcumin possesses such a low bioavailability. Addressing this problem, targeted and triggered drug delivery systems accompanied by nanoparticle technology have emerged as prominent solutions [23]. Likewise, this study introduces emulsomes as a promising nanocarrier system suitable for the delivery of curcumin.

**Figure 1:**
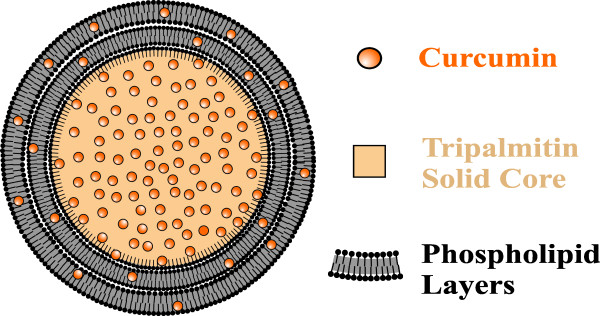
**Schematic drawing of CurcuEmulsome.** CurcuEmulsome is composed of a solid tripalmitin core surrounded by phospholipid multi-layers. The lipophilic load, i.e. curcumin, can locate itself in the inner core, as well as inside the phospholipid layers of the nanocarrier.

Emulsomes are biocompatible vesicular systems comprising of a solid fat core surrounded by phospholipid multi-layers (Figure 1) [27]. Due to the solid core, emulsomes can entrap higher amounts of lipophilic drug compounds with a prolonged release time compared to emulsion formulations possessing a liquid core [27-29]. Composed of fat and lipids, emulsomes are biocompatible. These characteristic properties make emulsomes to promising candidates for poorly water-soluble therapeutic agents such as curcumin.

As recently demonstrated, the assembly of phospholipids and triglycerides to form a stable dispersed emulsomes can be prepared by a dehydration-rehydration process followed by temperature-controlled extrusion [30]. In the present study, curcumin-emulsome nanoformulations, or so-called CurcuEmulsomes, were formulated using the same methodology, and characterized with respect to their structural and biophysical properties. HepG2 cell line was used as the *in vitro* cellular model to study cellular uptake of CurcuEmulsomes and to evaluate the biological effects of the incorporated curcumin on cellular morphology, as well as viability, compared to its free form. Cell cycle studies were performed to study CurcuEmulsome's effect on cell proliferation and implicitly to verify the incident of prolonged release of curcumin in biological level.

## Results

### CurcuEmulsome nanoformulation

**Figure 2:**
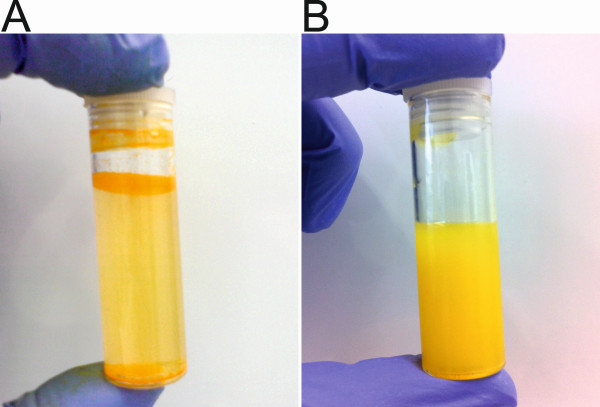
**Enhanced solubility. (A)** Free curcumin is poorly soluble in water and macroscopic flakes are visible especially on the top and the bottom of the bottle. **(B)** Curcumin incorporated into CurcuEmulsomes, in contrast, is homogeneously dispersed in water.

The structural design of CurcuEmulsomes enables curcumin to be localized in the inner solid tripalmitin core, as well as inside the phospholipid layers surrounding and stabilizing the nanocarrier (Figure 1). In contrast to free curcumin, poorly soluble in water (Figure 2A), curcumin incorporated into CurcuEmulsomes is a colloidal solution (Figure 2B). Forming an intensive turbid suspension, CurcuEmulsomes achieved curcumin concentrations up to 0.11 mg/ml (in range of 0.07-0.11 mg/ml) as estimated by absorbance measurements, where the latter value corresponds to a 10,000-fold increase in solubility of curcumin (i.e. ≈11 ng/ml [22]).

The aforementioned values correspond to the concentrations of curcumin incorporated into CurcuEmulsomes, as unincorporated curcumin in the solution was already spin down after a centrifugation process. Spin-down resulted in recovery of incorporated curcumin – corresponding to 90% of total - within the supernatant containing CurcuEmulsomes, indicating that a stable incorporation was achieved.

### Particular characterization of CurcuEmulsomes

**Figure 3:**
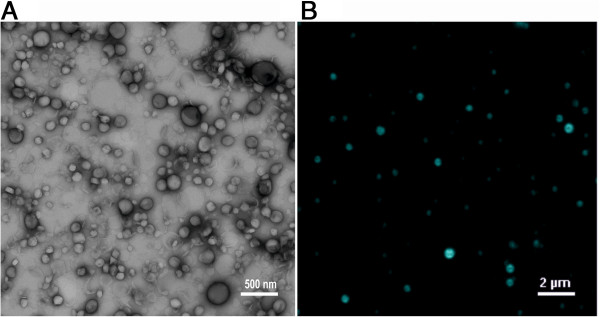
**Particular analysis of CurcuEmulsomes. A)** Transmission electron micrograph demonstrates CurcuEmulsomes are spherical in shape and have a diameter in range of 50–350 nm. **B)** Fluorescence microscopy image does not only confirm the incorporation of curcumin into CurcuEmulsomes, but also verifies the high-level dispersity of CurcuEmulsomes in water. Bars correspond to 500 nm and 2 μm, respectively.

TEM micrographs verified that CurcuEmulsomes are spherical in shape, and hence, similar in size and morphology to empty emulsomes (Figure 3A). DLS analysis with Zetasizer Nano ZS (Malvern Instruments Ltd, UK) determined the average diameter of ten distinct CurcuEmulsome formulations as 286 ± 27 nm (polydispersity index of 0.34; conductivity of 0.17 ± 0.01 mS/cm) - where the plus-minus sign indicates the margin of average size of numerous CurcuEmulsome formulations made of the same composition. Consistent with this value, the mean diameter of empty emulsomes was previously reported to be 297 ± 28 nm [30]. In addition, zeta potential of CurcuEmulsomes (37 ± 8 mV) is comparable to that of empty emulsomes (37 ± 7 mV) [30]. With the aid of the auto-fluorescence properties of curcumin, it was possible to evidence the incorporation of curcumin into emulsomes and that the prepared nanocarrier system is a stable dispersed formulation in water (Figure 3B). Consequently, the presented data declines any significant influence of incorporated drug neither on the size nor on the surface potential of the nanocarrier, which could further affect the particular dispersity of the nanocarrier in water.

### Tautomeric curcumin incorporated into CurcuEmulsomes in its enol form

**Figure 4:**
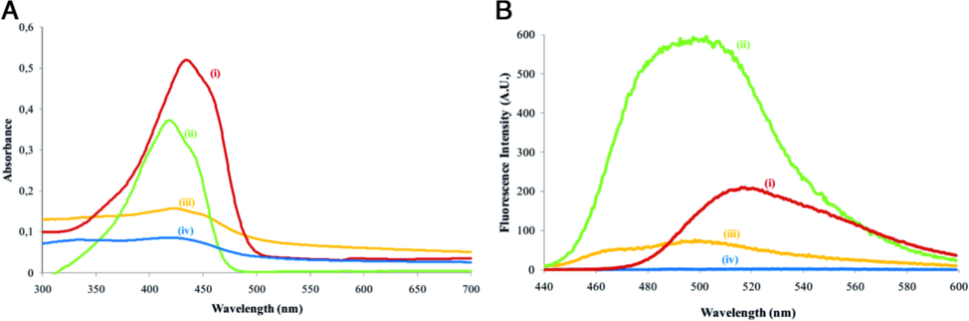
**Spectral analysis of CurcuEmulsomes. (A)** UV–vis absorbance spectra (excitation wavelength: 420 nm) and **(B)** emission spectra of curcumin in various forms: (i) curcumin in DMSO (red), (ii) curcumin in chloroform (green), (iii) curcuemulsomes in water (1:100 diluted) (yellow), and (iv) curcumin in water (blue).

Curcumin is a yellow-colored tautomeric compound that, upon dissolution in an organic solvent, absorbs light in the visible wavelength range [4]. In nonpolar, i.e. aprotic solvents such as chloroform, the spectrum displays vibronic structure with λ_max_ near 420 nm. This feature corresponds to the fully conjugated form of the protonated enol [31]. In polar protic solvents such as DMSO, the vibronic features are no longer resolved, and hence, the molar absorptivity decreases as solvent polarity increases resulting in λ_max_ shifts to nearly 430 nm [31]. In agreement with this [4,32,33], the UV–vis spectrum of CurcuEmulsomes displayed the same λ_max_ as curcumin in chloroform (420 nm), and differed from λ_max_ of curcumin dissolved in DMSO (Figure 4A). Hence, curcumin incorporated in CurcuEmulsomes is evidently in its fully conjugated protonated enol form.

Like the absorbance spectrum, the emission spectrum of CurcuEmulsomes pursued that of curcumin in chloroform and showed a λ_max_ at 500 nm (Figure 4B). Excitated at 420 nm, free curcumin in DMSO showed an emission peak centered at 520 nm and curcumin in water did not fluoresce.

### Curcumin composition inside CurcuEmulsomes

**Figure 5:**
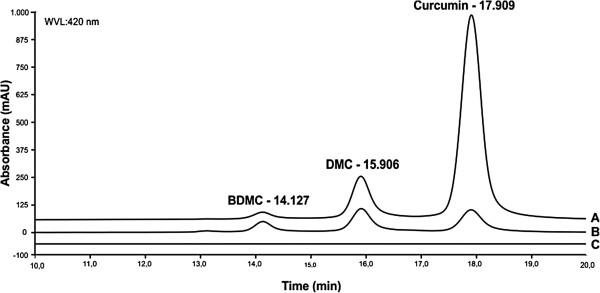
**Compositional analysis of CurcuEmulsomes.** HPLC chromatograms show the compositional distribution of curcumin and its analogues in **(A)** turmeric extract, **(B)** CurcuEmulsomes and **(C)** empty emulsomes. The HPLC data discloses that the composition of turmeric inside the nanocarrier is different than the one fed to the system. The plane curve of empty emulsomes declines occurrence of any interference of lipid composition of CurcuEmulsomes to the analysis.

Since turmeric as a mixture was demonstrated to have the same inhibitory effect as pure curcumin [8,9], curcumin was used as purchased without any further purification. Therefore, the turmeric fed to the system contained all three analogues, i.e. curcumin, DMC and BDMC. HPLC analysis showed that the turmeric extract consisted of 78.1% curcumin, 17.7% DMC and 4.1% BDMC (Figure 5A), whereas CurcuEmulsomes comprised of 40.8% curcumin, 40.3% DMC and 16.8% BDMC (Figure 5B). As curcumin analogues were the only substances in CurcuEmulsomes raising a peak at 420 nm, empty emulsomes did not show any peak in HPLC analysis (Figure 5C).

### Effect of CurcuEmulsomes on HepG2 cell viability

**Figure 6:**
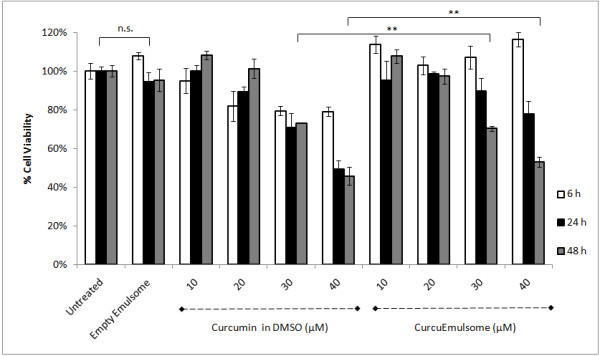
**Cell viability profile of HepG2 cells treated with curcumin and CurcuEmulsomes.** Cytotoxicity of CurcuEmulsomes, as well as free curcumin (in DMSO) and empty emulsomes, to HepG2 cells were investigated at various concentrations for 6, 24 and 48 hours compared to untreated cells. Cell viabilities are given in percentages relative to untreated cells. n.s., not significant; **comparable after 48 hours.

Previous studies demonstrated that 10–50 μM curcumin induces cell death primarily through apoptosis [25]. Within this range, HepG2 cells were treated with CurcuEmulsomes and free curcumin (in DMSO) of the same concentrations, respectively. After treatment for 6, 24 and 48 hours, the cell viability was determined with CellTiter-Blue assay. As shown in Figure 6, CurcuEmulsomes showed no significant cytotoxicity until 24 hours, in contrast to free curcumin which demonstrated significant toxicity especially in the early stage, i.e. after 6 hours. Nonetheless, on the long terms, incorporated curcumin preserved its biological activity, and thus, acted as efficient as free curcumin. Accordingly, after 48 hours 30 μM CurcuEmulsome lowered the viability of HepG2 to approximately 70%, 40 μM CurcuEmulsome to approximately 50%, same percentages as observed with free curcumin (Figure 6). In contrary, empty emulsomes showed no significant effect on HepG2 cell viability.

It is also important to mention that the viabilities recorded over 100% (Figure 6) might be due to the physical interference of the CurcuEmulsomes (not supported by our data since also the cells treated with curcumin at low doses showed the same response), as well as due to the changes in cellular activities involved in redox reactions in response to curcumin and CurcuEmulsomes, as CellTiter-Blue is a fluorescent assay used to measure cell viability via non-specific redox enzyme activity [34]. Therefore, although the latter hypothesis is likely to be the case, the complete clarification merits further study.

**Figure 7:**
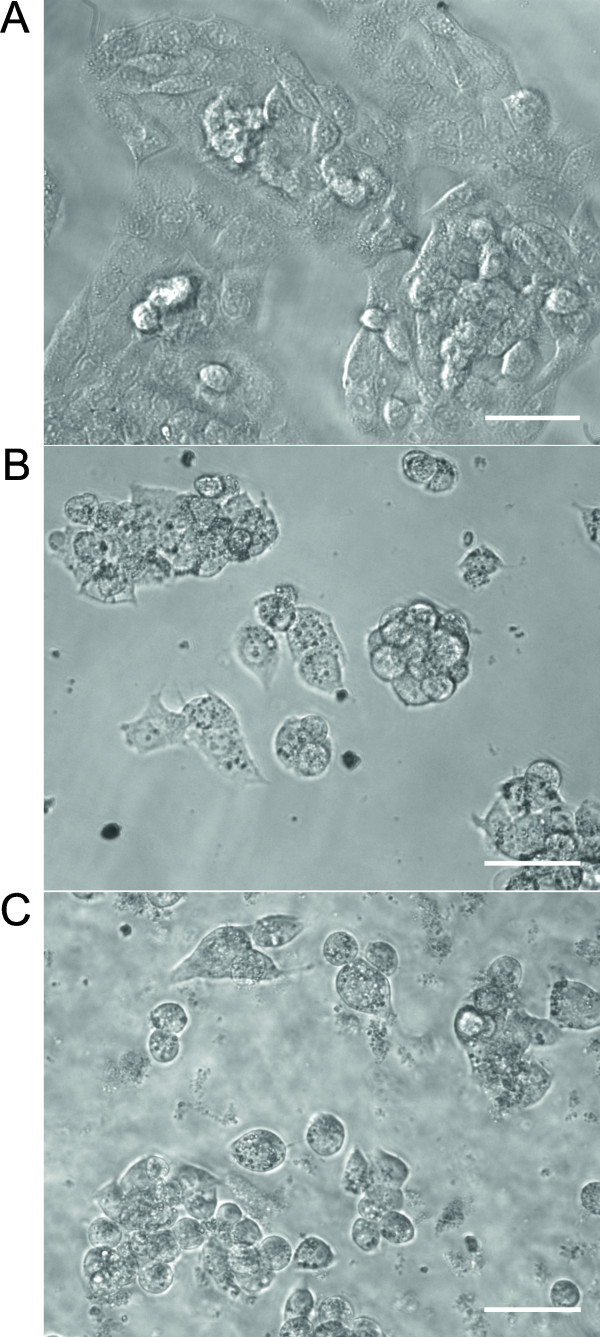
**Effect of CurcuEmulsome on cell morphology. (A)** Untreated HepG2 cells preserved their flattened morphology throughout the study, whereas cells treated with **(B)** curcumin or **(C)** CurcuEmulsomes (both 30 μM) showed a round shape after 24 hours of exposure. Bars correspond to 50 μm.

Considering interference with cellular adhesion, curcumin and CurcuEmulsomes caused also morphological changes in HepG2 cells. Cells treated with free curcumin and CurcuEmulsomes showed a round shape whereas untreated cells preserved their flattened morphology (Figure 7).

### Uptake of CurcuEmulsomes by HepG2 cells

**Figure 8:**
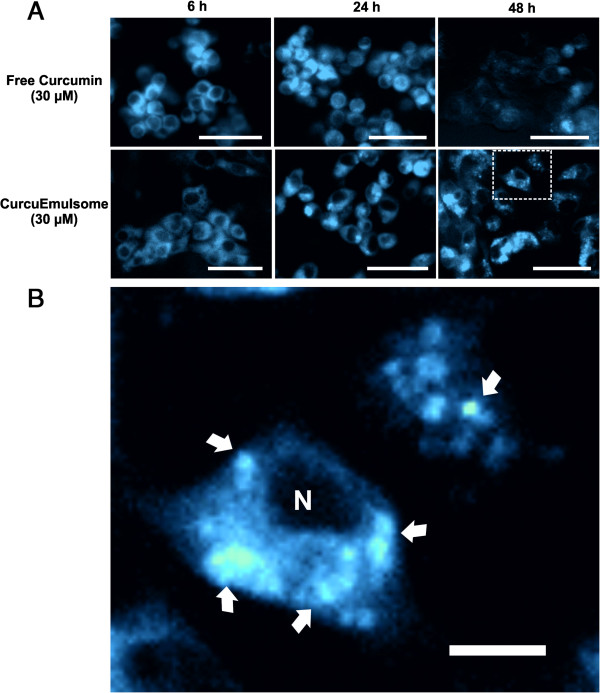
**Cellular uptake of CurcuEmulsomes by HepG2 cells. (A)** Cells were treated with 30 μM curcumin dissolved in DMSO, or incorporated into CurcuEmulsomes in 10% FCS MEM medium for 6, 24 and 48 hours. (Bars correspond to 50 μm). **(B)** Inset image of cells treated with CurcuEmulsomes (30 μM) for 48 h. Arrows indicate CurcuEmulsome accumulations upon cellular uptake. “N” indicates the cell nucleus. (Bar corresponds to 10 μm).

The uptake of CurcuEmulsomes in HepG2 cells could be evaluated by fluorescence microscopy analysis by the auto-fluorescence of curcumin (Figure 8). As previously reported [19], the cellular uptake was observed to be concentration-dependent as each increase in concentration from 10 μM to 50 μM resulted in an increase in fluorescence intensity inside the cell (data not shown). Along the time of treatment, fluorescence microscopy analyses were performed sequentially after 6, 24 and 48 hours and information was collected regarding the stepwise uptake mechanism and localization of curcumin and CurcuEmulsomes in HepG2. Accordingly, the fluorescence signal was limited to the cellular membrane for the first 6 hours, and widen to the inner compartments of the cells after 24 hours (Figure 8A). In agreement with Kunwar *et al.* (2008) [13], curcumin primarily localized in the cell membrane and subsequently around the nucleus, most likely due to their compartmental lipophilic properties. Moreover, in agreement with Mohanty *et al.* (2010) [26], cells treated with free curcumin showed the maximal fluorescence intensity at 24 hours, which faded down significantly with time (Figure 8A). On the contrary, cells treated with CurcuEmulsomes did not exhibit any deterioration in the level of fluorescence intensity neither after 24 nor 48 hours. This was attributed to the enhanced stability as well as to the gradual release of curcumin incorporated into the solid tripalmitin core of the nanocarrier. Hence, encapsulated curcumin remained protected from hydrolysis, and upon release, its biological activity persisted alongside its fluorescence intensity for a longer period of time than free curcumin.

Previous thin-sectioning analysis of HepG2 cells treated with empty emulsomes demonstrated that emulsomes are internalized in the cell within endosomes [30], resulting in an accumulation of the nanocarrier inside the cell before any sufficient release of the load could occur. Confirming this, the present data verified accumulation of CurcuEmulsomes inside the cytoplasm. Highly fluorescent spherical regions were discovered inside the cells treated with CurcuEmulsomes, which are ascribed to endosomes internalizing the nanocarriers. As indicated by arrows (Figure 8B), these regions were only detected for the cells exposed to CurcuEmulsomes for 24 and 48 hours. This finding may explain why CurcuEmulsome caused cytotoxicity first after 24 hours.

### Effect of CurcuEmulsomes on cell cycle

**Figure 9:**
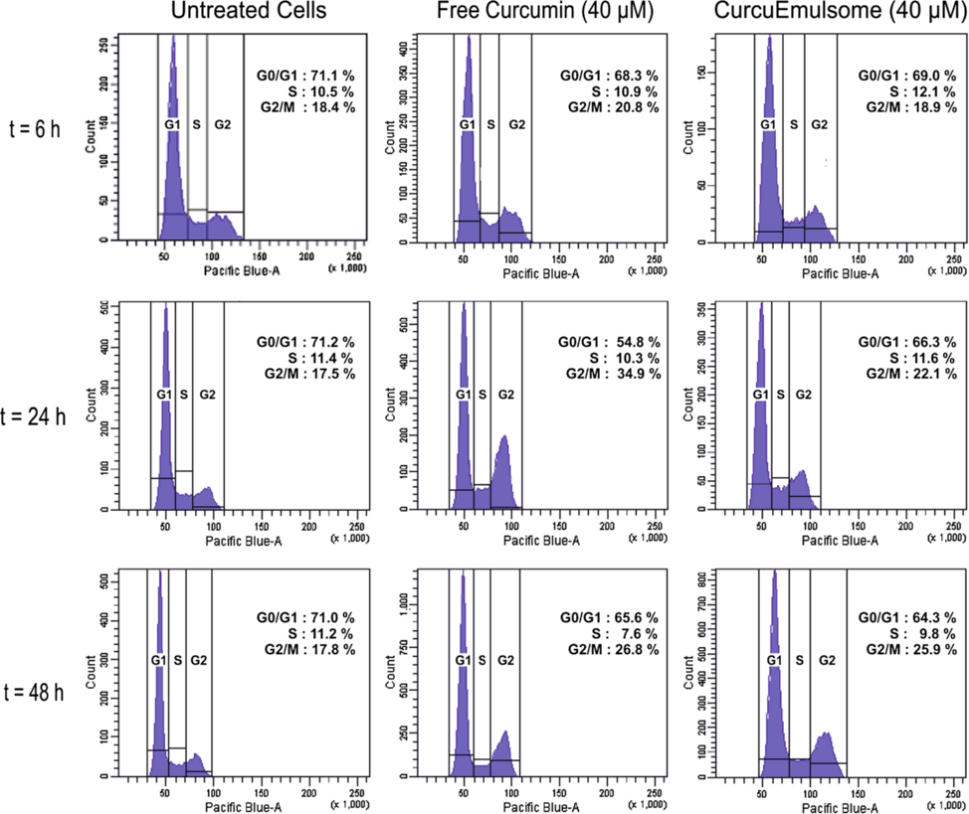
**Flow cytometric DNA histograms of HepG2 cells.** Effect of free curcumin and CurcuEmulsome on cell population of HepG2 cells was investigated for 6, 24 and 48 hours after exposure. Like free curcumin, CurcuEmulsomes induce G2/M cell cycle arrest. However, the expansion in population of cell at G2/M phase occurs after 48 hours, rather than 24 hours as for free curcumin. This delay is attributed to the gradual release of curcumin from the solid tripalmitin core of the nanocarrier inside the cell. Cells are labeled with DAPI for detection with Pacific Blue.

To explore the physiological effect of CurcuEmulsomes on cell proliferation, cell cycle analyses were performed on stable HepG2 cells with and without free curcumin or CurcuEmulsomes. Flow cytometry analysis demonstrated that HepG2 cells exposed to free curcumin (40 μM) for 24 hours were differentiated from untreated ones with a higher populations in the G2/M phase (35% instead of 18%) and with fewer fractions in the G0/G1 phase (55% instead of 71%; Figure 9). Compared to the control, this result suggested that curcumin inhibited the growth of HepG2 by causing cell-cycle arrest in the G2/M phase. Remarkably, G2/M phase arrest declined after reaching a peak at 24 hours indicating that thereafter free curcumin lost its activity and cells started recovery. On the contrary, CurcuEmulsome treatment at 40 μM resulted in a steady increase of cell population in G2/M phase from 19% to 22% and then to 26%, as population in G0/G1 phase decreases from 69% to 66% and then to 64%, from 6 to 24 hours and subsequently to 48 hours, respectively. At 48 hours, the cell cycle profiles of cells treated with curcumin and CurcuEmulsomes became comparable: around 26% of the cells in G2/M and 65% in G0/G1 phase (Figure 9). Cell cycle profiles of untreated cells remained unaltered throughout the experiment. Concisely, like free curcumin, CurcuEmulsome induced G2/M cell cycle arrest on HepG2 cells, but this was prolonged probably since curcumin was released inside the cell gradually over time.

### Effect of CurcuEmulsomes on apoptosis

**Figure 10:**
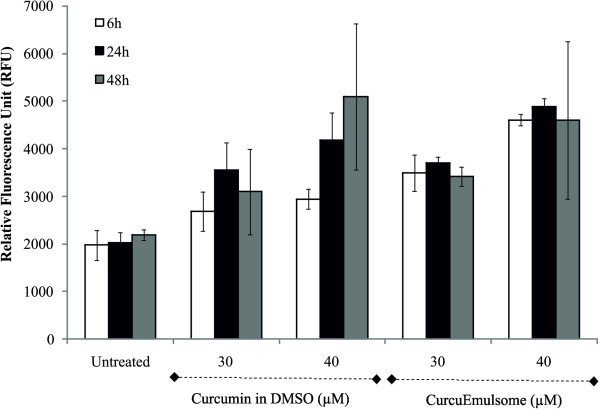
**Apoptotic activities of HepG2 cells treated with CurcuEmulsomes.** Apoptosis responses of HepG2 cells to free curcumin and CurcuEmulsomes were analyzed by a Caspase 3/7 activity assay. The level of fluorescence intensity corresponds to the level of apoptosis.

The apoptosis response of HepG2 to CurcuEmulsomes and free curcumin was analyzed by a Caspase 3/7 activity assay in which higher fluorescence intensities correspond to higher level of apoptosis. Like free curcumin, CurcuEmulsomes caused a concentration-dependent increase in apoptosis with comparable apoptotic activities at 24 and 48 hours (Figure 10). These results strongly suggested that the cytotoxicity of CurcuEmulsomes can be attributed to the induction of apoptosis and G2/M phase cell cycle arrest.

## Discussion

The results of this study indicate that CurcuEmulsomes can successfully entrap curcumin inside the inner solid matrix composed of tripalmitin surrounded by phospholipids. The stable formulations are spherical in shape and preserve the surface characteristics of the nanocarrier. Most important, the solubility of curcumin is increased up to 0.11 mg/ml by means of CurcuEmulsomes, corresponding to an improvement in solubility by 10,000 times. Thus CurcuEmulsomes can achieve the effective concentrations of curcumin (i.e. 10–50 μM) [25,26], and facilitate the delivery of bioactive molecules into the cell *in vitro*.

In the literature, various encapsulation approaches like diblock copolymers [35,36], hydrophobically modified starch [32], beta-casein micelles [37], lipid nanoemulsions [38], curcumin-rubusoside complexes [39], cyclodextrin assemblies [40,41], liposomes [42], curcumin-nanodisk [4] and polymeric NanoCurc™ formulations [43] have been successfully applied to increase the solubility and thereby the delivery of curcumin. Encapsulation of curcumin in a pluronic block copolymer showed not only anti-cancer activity comparable with free curcumin, but also demonstrated a slow and sustained release of curcumin [36]. Therefore, the aforementioned approaches, as well as CurcuEmulsomes, look promising to enable the effective use of curcumin in medical applications.

However, having partially the characteristics of both liposomes and emulsions, CurcuEmulsome approach possesses certain advantages over its alternatives. Like liposomes, emulsomes are stabilized by phospholipid (multi-)layers as outermost structure, and thus, there is no need for surfactants stabilizing the nanoformulation. This endows emulsomes high degree of biocompatibility at therapeutic applications. More detailed, in the absence of any synthetic surfactants such as poloxamers, polysorbates or doxycholate, the use of emulsomes as a drug delivery system has demonstrable advantages, particularly for parenteral administration of poorly water-soluble lipophilic drugs [27], such as curcumin. Alternatively, due to their colloidal nature, emulsomes can be passively taken up from the blood stream by macrophages of the liver and spleen after intravenous or intracardiac administration as demonstrated in early *in vivo* studies [44,45].

On the other hand, unlike lipid emulsions having a fluid core, emulsomes with a solid fat core can prolong the release of incorporated drugs - a property similar to polymeric nanoparticles [28,46]. As previously demonstrated, zidovudine-emulsome formulations displayed a slow drug release profile *in vivo* (12–15% after 24 h) and prolonged the action at comparatively low drug doses [29]. Therefore, the developed CurcuEmulsomes would be expected not only to circumvent the problems of low solubility and rapid elimination, but also to modify the drug release profile thereafter, due to the presence of curcumin in the internal solid lipid core.

Finally, having an analogous surface as liposomes [47], CurcuEmulsomes can further be tailored to fulfill specific requirements such as longer blood circulation or to enable cell targeting and active drug delivery. For instance, Gill *et al.* (2011) coated emulsomes with O-palmitoyl amylopectin [48], whereas Pal *et al.* (2012) coated them with O-palmitoyl mannan both with the aim of developing macrophage targeted systems [45]. In a recent study, we showed that crystalline bacterial cell surface layer (S-layer) proteins are capable to coat emulsomes and modify their entire surface characteristics [30], e.g. by altering zeta-potential.

The colloidal characteristics of the emulsome evidence its robust character and indicate its potential in versatile use for lipophilic therapeutic agents other than curcumin. As previously reported [28-30], the size of emulsomes (a mean diameter of 286 nm, Figure 3) is predominantly determined by the phospholipid to tripalmitin ratio, and evidently, incorporation of curcumin did not influence neither particle size nor zeta potential characteristics. Moreover, the particle sizes can be tuned by altering the phospholipid to solid lipid ratio [29].

Although curcumin, DMC and BDMC show only very small chemical modifications with respect to their number of methoxy groups, a decrease in hydrophobicity in the order of curcumin > DMC > BDMC is known [49]. Therefore, a shift in the ratio of the analogues inside the lipophilic fat core should be expected, but not in terms of a relative decrease of curcumin compared to DMC and BDMC (Figure 5). Hence, this result contradicts with the relative hydrophobicity of the analogues, as well as the findings of Rungphanichkul *et al.* (2011), where encapsulation of curcuminoids in non-ionic surfactant based liposomes, so-called niosomes, favored the incorporation of curcumin rather than its analogues [50]. Although some thermodynamic parameters such as the polarity, as well as the molecular electrostatic interactions of curcuminoids with charged groups of lipid compounds, such as hexadecylamine, are thought to play a role in this selective incorporation process, the complete clarification of this finding merits further study.

Biological efficacy of CurcuEmulsomes was studied *in vitro* on HepG2 cell line model. In line with earlier studies on emulsomes [29], the delay in cytotoxicity is attributed to the slow release of curcumin entrapped inside the solid core of emulsomes. Hence, on the short terms the cytotoxic effect of CurcuEmulsomes remains limited. Nevertheless, CurcuEmulsomes displayed prolonged biological activity and acted as efficiently as free curcumin on long terms (Figure 6).

Like free curcumin, CurcuEmulsomes caused morphological changes in HepG2 cells where treated cells distinguished from untreated ones by their round shape. Based on AFM studies, Jiang *et al.* (2012) demonstrated the effect of curcumin on cytoskeletal arrangement of HepG2 cells and, combined with flow cytometric analysis, correlated this morphological effect with the upregulated expression of tubulin [21]. The latter caused disorganization of the well-organized, filamentous network of healthy cells as deduced from the adopted round shape. Therefore, delivering curcumin into the cell, CurcuEmulsomes must be initiating the same effect (Figure 7).

Indicating for an enhanced stability, fluorescence images demonstrated that incorporated curcumin preserve its fluorescence intensity for longer times compared to free curcumin (Figure 8A). Parallel to our previous cross-sectional analysis of cells treated with empty emulsomes [30], the fluorescence microscopic data verified the accumulation of CurcuEmulsomes inside the cytoplasm upon their uptake by the cell (Figure 8B). Accordingly, CurcuEmulsomes accumulate inside the cell before any sufficient release of the load could occur. This finding may explain why CurcuEmulsomes caused cytotoxicity only after 24 hours (Figure 6).

Cell cycle analysis demonstrated that CurcuEmulsomes cause a prolonged induction of G2/M cell cycle arrest where the peak of G2/M phase rose steadily from 6 to 48 hours (Figure 9). In the contrary, free curcumin results in a sharp increase after 24 hours which declined after 48 hours. These findings, in line with cytotoxicity data, corroborate the slow and sustained release of curcumin from CurcuEmulsomes into the cells. Cell cycle analyses were only performed for 48 hours because the low viability profiles of treated HepG2 cells (Figure 6) did not allow longer investigations. However, speculatively, a further increase in G2/M phase arrest might be predicted due to the slow release profile of emulsomes.

## Conclusions

Introducing a new nanocarrier system for curcumin, the present study illustrates the particular characteristics of CurcuEmulsomes and investigates the delivery of curcumin into the cell *in vitro*, where HepG2 cell line is used as a model. In summary it may be concluded that i) curcumin can be incorporated into the emulsomes, ii) the incorporation enhances the poor water solubility of this bioactive polyphenol, iii) upon incorporation, biological activity as well as fluorescence integrity of curcumin is preserved, iv) delivered within a solid lipid core, curcumin is gradually released into the cell, thereby resulting in prolonged cytotoxicity and cell cycle arrest on HepG2, v) due to its prolonged activity, the incorporated curcumin acts, on long terms, as efficient as free curcumin dissolved in organic solvent. Consequently, enabling curcumin to reach its effective concentrations inside the cell, the presented approach may allow therapeutic applications of curcumin, and with future perspectives, provide an alternative platform for the delivery of hydrophobic bioactive agents whose medical use is otherwise limited.

## Methods

### Materials

Curcumin, glyceryl tripalmitate (tripalmitin, purity ≥99%), 1,2-dipalmitoyl-rac-glycero-3-phosphatidylcholine (DPPC, 99%), glutaraldehyde solution (50%), glycerol (99%) and Dulbecco’s Phosphate Buffered Saline (PBS, 10X) were purchased from Sigma-Aldrich GmbH, Germany. Hexadecylamine (HDA, ≥99%), uranyl acetate dehydrate (≥98%), methanol (99.5%) and chloroform (≥99.8%) were obtained from Fluka Chemika, Germany. Cholesterol (>98%) was purchased from Avanti Polar-Lipids, US. Dimethyl sulfoxide (DMSO) was purchased from Riedel-de Haën (Sigma Aldrich, Germany). 4′,6-Diamidino-2-phenylindole dihydrochloride (DAPI) was purchased from AppliChem GmbH, Germany. All chemicals were used as purchased without any further purification.

### Cell line

HepG2 (human liver hepatocellular carcinoma cell line) was obtained from American Type Culture Collection (Rockville, MD; ATCC HB-8065). HepG2 cells were cultured in Minimal Essential Medium (MEM) with Earlea’s Salts medium (PAA, Pasching, Austria), supplemented with 10% fetal calf serum (FCS) albumin and 1% antibiotic/antimycotic (both PAA) at 37°C in a humidified atmosphere of 5% CO_2_ and 95% air.

### CurcuEmulsome preparation

Curcumin and tripalmitin with a weight ratio of 2:5 were dissolved in chloroform. DPPC, cholesterol and HDA with a molar ratio of 10:5:4 were dissolved separately in chloroform. Both lipid solutions were mixed and the organic solvent was completely removed using a rotary evaporator (Rotavapor R-215, Büchi, Switzerland) under reduced pressure at 474 mbar and 60°C. The formed dry film was hydrated with MilliQ water, the temperature was set to 80°C and the solution was rotated until the lipid film was resuspended. The obtained product was homogenized by high pressure extrusion system with heating control (Avestin Liposo Fast LF-50, Ottawa, Canada). At a temperature of 66°C and under an overpressure above 10 bars, the solution was passed multiple times through 800-nm and 400-nm polycarbonate filters (Nucleopore Track-Etch Membrane, Whatman, UK). Immediately after extrusion, the obtained emulsome suspension was placed on ice for 10 min. CurcuEmulsome preparations were centrifuged at 13,200 rpm (16,100 g) for 10 minutes to spin down unincorporated curcumin. The CurcuEmulsome suspension, i.e. the supernatant, was stored at 4°C until further characterization and cell culture studies. Empty emulsomes were prepared as described above but without curcumin [30].

### Quantification of curcumin by absorbance measurements

A 1 mg/ml stock solution of curcumin was prepared in DMSO. A standard curve, generated by successive dilution of the stock solution (5, 10, 20, 50, 100 μg/ml) in a 96-well microplate (Cellstar, Greiner Bio-One GmbH, Frickenhausen, Germany), was used to determine curcumin concentrations in samples prepared by dilution of CurcuEmulsome suspension 1:10 in DMSO. Sample absorbance was measured at 430 nm on Infinite F200 plate reader (TECAN, Austria).

### Compositional analysis of CurcuEmulsomes

The composition of CurcuEmulsomes was determined by HPLC. CurcuEmulsome formulation was dissolved in methanol to disrupt its structure. The sample was subjected to sonication for 3 min at 170 W (Transsonic T 460, Elma, Germany) followed by centrifugation at 14,680 rpm (20,238 g) for 10 min at 25°C (Centrifuge 5424, Eppendorf, Germany). The clear supernatant was analyzed using reverse phase isocratic mode (RP-HPLC) on Summit HPLC systems (Dixon/ThermoFisher Scientific, Germany). In brief, 10 μl of the sample was injected automatically in the injection port and analyzed on C18 column (Nucleosil 120-3C18, 150×4 mm, Macherey-Nagel, Germany) with the mobile phase consisting of acetonitrile and 2% acetic acid (40:60, v/v) at 33°C [51]. The amount of curcumin was quantified by UV detection at 420 nm with UV/VIS-Detector UVD 170U/340U (Dionex, Germany). The compositional distribution of curcumin in the sample was determined from the peak area correlated with the standard curve. The total HPLC analysis time was 20 min per sample, with curcumin, DMC and BDMC eluting at retention times of 17.3, 15.4 and 13.7 min, respectively.

### In vitro cytotoxicity assay

Cytotoxicity of CurcuEmulsomes was examined by CellTiter-Blue Cell Viability Assay (Promega, Germany) as described previously by Ucisik *et al.* (2013) [30]. Briefly, HepG2 cells were seeded in 96-well microtiter plates at a density of 10,000 cells per well in a final volume of 300-μL culture medium. After 24 h, the cell culture media were aspirated and the cells were treated with 100-μl culture medium containing free curcumin (in DMSO) or CurcuEmulsomes at various concentrations. Other cells were left untreated as negative control. DMSO content in total cell medium was kept below 0.15% to avoid any influence of DMSO to HepG2. Fluorescence intensity of cells was recorded using Infinite F200 plate reader (TECAN, Austria) with a 560(20)_Ex_/595(35)_Em_ fluorescence intensity filter (TECAN, Austria).

### Cell cycle analysis

HepG2 cells were seeded in cell culture flasks at a density of 500,000 cells per 25 cm^2^. After two days of incubation cell medium was changed with 5-ml culture medium containing free curcumin (40 μM) or CurcuEmulsome (40 μM). Other cells were left untreated as negative control. Cells were harvested, washed three times with PBS, then counted and resuspended in PBS at concentration of 1×10^6^ cells/ml. Of each sample 3×10^5^ cells (300 μl) were stained with 2 μg/ml DAPI in methanol for 15 min at room temperature in the dark. Subsequently, cells were centrifuged at 1250 rpm for 5 min, resuspended in ice-cold FACS buffer (Becton Dickinson (BD), Austria) and immediately analyzed via a FACSCanto II Flow Cytometer equipped with a BD FACSDiva acquisition and analysis program (BD, Austria). Samples, stained with DAPI, were excited with a 405-nm blue laser and the emitted light in the region of 450(50) nm (Pacific Blue) was recorded. Data from at least 10,000 cell counts were collected for each data file. Gating was set properly to exclude cell debris, cell doublets, and cell clumps.

### Apoptosis test

The apoptosis response of HepG2 cells to CurcuEmulsomes and free curcumin in DMSO were analyzed by Cell Meter Caspase 3/7 Activity Apoptosis Red Fluorescence Assay Kit (AAT Bioquest, Biomol, Germany). Briefly, HepG2 cells were seeded in 96-well microtiter plates at a density of 10,000 cells per well in a final volume of 100-μL culture medium. After 24 h, the cell culture media were aspirated and the cells were treated with a medium containing free curcumin (in DMSO) or CurcuEmulsomes at various concentrations for 6, 24 and 48 h. Other cells were left untreated as negative control. At the time of analysis, the medium was replaced and equal volume of Z-DEVD-ProRed™ Reagent Assay was added to each well. Following incubation of cells at room temperature for 70 min in the dark, the fluorescence intensity at Ex/Em = 535(25)/635(35) nm was monitored by Infinite F200 plate reader.

### Ultraviolet–visible (UV–vis) absorbance spectroscopy

UV–vis absorbance spectroscopy was performed on a U-2900 UV/Vis spectrophotometer (Hitachi, Japan). Samples were scanned from 300 to 700 nm.

### Fluorescence spectroscopy

Fluorescence spectra were obtained on a Perkin Elmer LS 55 luminescence spectrometer (Perkin Elmer, UK). Curcumin samples were excited at 420 nm, and emission was monitored from 430 to 600 nm (2.5-nm slit width).

### Dynamic and phase analysis light scattering

Diluted in 1 mM KCl solution (pH 6.3) CurcuEmulsomes with a final DPPC concentration of 4 μg/ml were analyzed by the Zetasizer Nano ZS (Malvern Instruments Ltd, UK) for their particle size distribution (Dynamic Light Scattering; DLS) and zeta potential characteristics (Phase Analysis Light Scattering; M3-PALS) as previously described [30].

### Electron microscopy

The shape and the integrity of CurcuEmulsomes were analyzed by a FEI Tecnai G^2^ 20 Transmission Electron Microscope (TEM) at 120 kV equipped with FEI Eagle 4 k camera (FEI Europe, The Netherlands) after negative staining with uranyl acetate (1% in MilliQ) as described by Ucisik *et al.* (2013) [30].

### Fluorescence microscopy

Nikon Eclipse TE2000-S fluorescence microscope (Nikon, Melville, NY) was used to visualize the samples. The images of cell cultures were taken with 20× and 40× objectives, and those of CurcuEmulsomes preparation with 100x oil immersion objective. Curcumin incorporated in emulsomes was detected using a Cyan Fluorescent Protein (CFP)-Fluorescence filter (Excitation at 436/20 nm; Emission at 480/40 nm).

## Competing interests

The authors declare that they have no competing interests.

## Authors' contributions

MHU synthesized CurcuEmulsomes and performed the characterization experiments. MHU and SK executed *in vitro* cell culture studies. MHU and SK conceived the idea of CurcuEmulsomes. SK, BS and UBS guided the conduct of studies, supervised data analysis, and authored the manuscript. All authors have read and approved the manuscript.

## Authors’ information

This study embodies a part of MHU’s PhD study.
